# Influence of copper(I) nicotinate complex on the Notch1 signaling pathway in triple negative breast cancer cell lines

**DOI:** 10.1038/s41598-024-52952-1

**Published:** 2024-01-30

**Authors:** Mohamed A. Abdel-Mohsen, Asmaa M. Badawy, Morsy A. Abu-Youssef, Mona A. Yehia, Lobna D. Abou Shamaa, Shymaa Abdullah Mohamed

**Affiliations:** 1 Applied Medical Chemistry Department, Medical Research Institute, Alexandria University, Alexandria, Egypt; 2 Chemistry Department, Faculty of Science, Alexandria University, Alexandria, Egypt; 3 Histochemistry and Cell Biology Department, Medical Research Institute, Alexandria University, Alexandria, Egypt; 4 Immunology and Allergy Department, Medical Research Institute, Alexandria University, Alexandria, Egypt; 5 Molecular Biology Unit, Medical Technology Center and Applied Medical Chemistry Department, Medical Research Institute, Alexandria University, Alexandria, Egypt

**Keywords:** Biochemistry, Cancer, Drug discovery, Molecular biology

## Abstract

Triple negative breast cancer (TNBC) is a subtype of breast cancer which is characterized by its aggressiveness, poor and short overall survival. In this concept, there is a growing demand for metal-based compounds in TNBC therapy as copper complex that have a less toxic effect on normal cells and could stimulate apoptotic cell death. Additionally, Notch1 signaling pathway has received great attention as one of the most important potential targets for developing a novel therapeutic strategy. The present study is an attempt to assess the promising chemotherapeutic activities of copper(I) nicotinate (CNC) through its impact on the expression of downstream genes of Notch1 signaling pathway and the cell fate of TNBC. The co-treatment of TNBC cells with doxorubicin (Doxo) and CNC was also investigated. To approach the objective of the present study, TNBC cell lines; HCC1806 and MDAMB231, were utilized. MTT assay was used to determine the IC_50_ values of CNC and Doxo. After treatment, microtubule-associated protein light chain3 (LC3) were determined by flow cytometry. Additionally, qRT-PCR technique was used to detect the changes in genes levels that are involved Notch1 signaling pathway. Moreover, autophagosomes were monitored and imaged by Transmission electron microscopy. Treatment of TNBC cells with CNC modulated Notch1 signaling pathway in different manners with respect to the type of cells and the applied dose of CNC. The observed effects of CNC may reflect the possible anti-cancer activities of CNC in both types of TNBC. However, cell type and CNC dose should be considered.

## Introduction

Based on specific genes expression patterns, breast cancers are categorized into five intrinsic or molecular subtypes. Among the intrinsic subtypes, triple-negative breast cancer (TNBC) accounts for 12–20% of breast cancers types^1^. In Egypt, according to cancer 12-years registry, National Cancer Institute, Cairo University; TNBC constitutes about 13.5% of breast cancer^2^. TNBC has drawn specific attention due to the lack of expression of all three receptors, ER, PR, and Her2. Thus, it cannot be treated using anti-estrogen hormonal therapies^3^. Thus, developing a more effective therapeutic method for TNBC patients is necessary.

However, inappropriate activation of signal transduction, and indeed the inability to respond to negative signals, is a hallmark of cancer. Deeper insights into the pertinent and targetable signaling pathways in TNBC may offer new avenues for therapeutic intervention. Notch signaling pathway is aberrantly activated in breast cancer, with increased Notch intracellular domain accumulation and target gene expression detected in a range of breast cancer cell lines and primary samples^4–6^. Over-expressions of Notch receptors and ligands have been reported in breast tumors, and correlated with poorer patient prognosis^7^. Aberrant Notch signaling has also been extensively linked to TNBC subtype. Where, Notch receptor over-expression is correlated with the aggressive, metastatic and therapy resistance phenotype characteristic of TNBC^8,9^.

Doxorubicin is regarded as the most therapeutic active agent available for TNBC treatment. However, the development of drug resistance and toxicity limits its effectiveness. Thus, developing novel strategies for TNBC treatment remains a significant challenge. In this aspect, a great deal of research has been conducted on the therapeutic applications of metal-based complexes and there is a growing demand for metal-based compounds in cancer therapy. Copper complexes were found to have a less toxic effect on normal cells and could stimulate apoptotic cell death^10,11^ and have shown anticancer activity due to their ability to produce reactive oxygen species^12^. Generally, it should be noted that trafficking of copper(I) complexes in cells is preferable than copper(II) compound^13^. Evidentially, copper(I) nicotinate complex (CNC) displayed several anti-tumor activities in TNBC including pro-apoptosis, anti-autophagy as well as anti-angiogenesis^11^. Therefore, and based on the previously mentioned observations concerning CNC, the present study was undertaken to reveal the possible utilization of Notch1 signaling pathway as a molecular target in the therapy of TNBC with CNC.

## Subjects and methods

### Drugs and chemicals

3-(4,5-dimethylthiazol-2-yl)-2, 5-diphenyltetrazolium bromide (MTT) was purchased from (Sigma-Aldrich Co., USA). Doxorubicin (Doxo) was purchased from the pharmacy. RNA was extracted using easy-spin™ Total RNA Extraction Kit while, gene expression profiles were done using HISenScript™ RH [-] cDNA Synthesis Kit and SYBR Green with low ROX (TOPreal™ qPCR 2X preMIX); all were purchased from, iNTRON Biotechnology. Fluorochrome-conjugated primary antibody of LC3-II was purchased from cusabio, USA. Dulbecco's Modified Eagle Medium (DMEM) was obtained from Lanza Bioproduct, Belgium, where fetal bovine serum (FBS) from Biowest, UK.

### Cell culture

TNBC cell lines, HCC1806 (ATCC®CRL-2335™) and MDA-MB-231 (ATCC®HTB-26™), were purchased from the American Type Culture Collection (ATCC, Manassas, VA, USA). Cells were maintained in DMEM supplemented with 10% FBS at 37 °C in 5% CO_2_ incubator_._

### Preparation of copper(I)-nicotinate complex (CNC)

The [Cu(I)-(nicotinic acid)2] + Cl- complex ‘CNC’ was synthesized as described by Gohar and Dratoviscky in 1975^14^ through the reaction of ethanolic solution of CuCl_2_-2H_2_O with nicotinic acid in the presence of “L + ascorbic” acid. Generally, 1.45* g* of nicotinic acid was dissolved in 50 mL boiling distilled water and then added to an ethanolic solution of CuCl_2_–2H2O (0.85* g*, 40 mL). After cooling the mixture, 0.5 g of “L + ascorbic” acid was added and left at room temperature until clear orange-red crystals were obtained. The crystals were filtered and washed with ethanol. The pure bright red needle crystals were examined by an infrared spectrum, which indicated that the chemical structure is “Cu (HNA)2] + Cl” confirming the chemical composition of Cu(I)-(nicotinic acid)_2_Cl-2H_2_O^15^.

### Cytotoxicity assay

**Figure 1:**
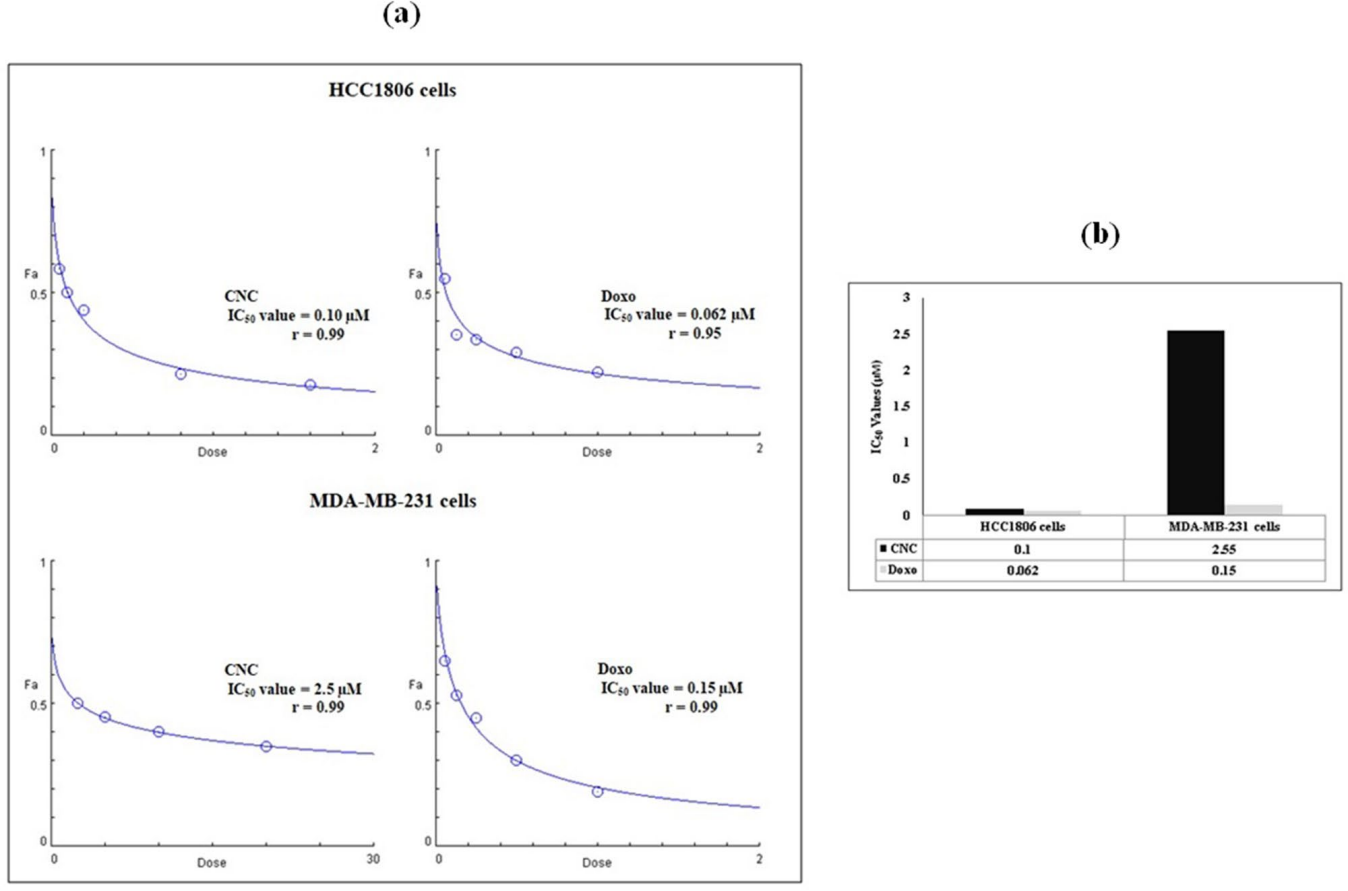
**(a)** Charts and **(b)** histograms showing the half maximal inhibitory concentration (IC_50_) values of CNC and Doxo on HCC1806 and MDA-MB231 cells. IC_50_ values showed that HCC1806 cells were more sensitive to CNC and Doxo than MDA-MBA231 cells. The IC_50_ values may reveal that HCC1806 cells are more sensitive to CNC and Doxo than MDA-MBA231 cells. **(c)** Cytotoxicity percentage (%) of IC_50_ values of DOXO and CNC against HCC1806 and MDA-MB231 cells. The data were analyzed with Mann Whitney U Test. Error bars represent mean ± SD. *P < 0.05 versus corresponding control.

To perform the Cytotoxicity test for HCC1806 and MDA-MB231 cells, 10,000 cells/well were seeded in 96-well plates and maintained at 37 °C in 5%CO_2_ incubator till cells reached 80% confluent according to the ATCC protocol. CNC and Doxo were added to appropriate wells at different concentrations and supplemented with complete media to a final volume of 200μL then incubated for 24 h.After incubation period, 10μL of MTT solution (5 mg/mL) was added to each well and incubated for 4 h at 37 °C. Control samples were setup (50 µL of cells and 50 µL MTT solution). After incubation time, media was removed and 100 µL of dimethylsulfoxide (DMSO) were added. Optical density was determined at 590 nm using ELISA reader (BioBioTek Company, UA). The cells were harvested and mixed in a 1:1 ratio with trypan blue for exclusion staining. The number of living cells was counted and IC_50_ values of CNC and Doxo were determined. Experiment was repeated three times for statistical analysis.IC_50_ values showed that HCC1806 cells are more sensitive to CNC and Doxo than MDA-MBA231 cells Fig. 1a, b.

### Design of experiment

HCC1806 or MDA-MB-231 cells were seeded in 12-well plates (3 × 10^4^/well in 1.5 mL) and incubated for 48 h at 37 °C in 5% CO_2_ incubator. For each cell line and after determination of IC_50_ values of CNC and Doxo, the experiment was designed as: CNC_50_ (treated cells with IC_50_ of CNC), CNC_10_ (treated cells with 10% IC_50_ of CNC), Doxo_50_ (treated cells with IC_50_ of Doxo), CNC_50_-Doxo (treated cells with IC_50_ of CNC and Doxo) and CNC_10_-Doxo (treated cells with 10% IC_50_ of CNC and IC_50_ of Doxo) groups in addition to untreated cells as control group. This experiment was repeated 3 times. After 24 h of treatments, the cell morphology alterations were examined under an inverted microscope with magnification of 200 × and then the cells were harvested for downstream analysis.

### Monitoring autophagy using transition electron microscopy

After treatment, collecting cells (1 × 10^6^) were washed with 0.5 ml 0.1 M phosphate buffered saline (PBS) and centrifuged at 300 x*g* for 5 min. Cells were fixed in 2% glutaraldehyde in 0.2 M HEPES, *p*H 7.4, at room temperature (R.T) for 2 h. Cell pellet was washed 3 times with 0.2 M HEPES, pH 7.4 and centrifuged at 300 *x*g for 5 min. The pellet was then fixed in 1% osmium tetroxide in water at room temperature for 1 h and then washed the cells 3 times with HEPES and centrifuged at 300 x*g* for 5 min. Then the pellet was stained in 2% uranyl acetate in water at room temperature, in the dark, for 1 h. After staining, dehydration at R.T was carried out using 70% ethanol for 15 min; 95% ethanol for 15 min; 100% ethanol, twice for 15 min and propylene oxide for 20 min. Then, the pellet was incubated in 100% propylene oxide resin at R.T overnight. After transferring the pellets to fresh resin in beem capsules and incubation at room temperature for 6 h, the block was polymerized at 60 °C for 2 days. Sections of 80 nm were prepared using diamond knife and post stained with 2% uranyl acetate in water for 15 min and 0.3% lead citrate for 3 min, according to standard grid staining procedures. Finally, observation was carried out using JEM-1400 plus electron microscope.

### LC3-II detection using flow cytometry

Harvested cells (1 × 10^6^) were fixed using 4% formaldehyde for 10 min at 37 °C. After centrifugation at 300 x*g* for 5 min cells were permeabilized by adding ice-cold 100% methanol slowly to pre-chilled cells, while gently vortexing, to a final concentration of 90% methanol and incubated for 30 min on ice. 2 mL of incubation buffer (10% fetal bovine serum in PBS) were added to cells and washed by centrifugationat 300 x*g* for 5 min. Cells were re-suspended in 100μL of fluorochrome-conjugated primary antibody and incubated for 1 h at R.T then washed by centrifugation in 2 mL incubation buffer. Cells were re-suspended in 0.5 mL 1 × PBS and analyzed on flow cytometer^16^.

### Real-time PCR analysis

Total RNA was extracted according to the instruction manufacture. 5 µg of extracted and purified RNA was used to synthesis cDNA. The cDNA was used for PCR amplification using specific pair of primers for the following genes; NOTCH1, JAG1and HES1 for studying the Notch1 signaling pathway and β-actin as a housekeeping gene. Table 1 PCR mixture was prepared with 10 μl of TOPreal™ master mix, 2 μl of cDNA and 0.0.5 μM of each primer and then the volume was completed up to 20 μl. PCR conditions were assessed as; on cycle of 94 °C for 5 min, 45 cycles of 94 °C for 30 s, 59 °C for 30 s and 72 °C for 1 min. qRT-PCR was assessed using Applied Biosystems™ 7500 Real-Time (Thermo-fisher Scientific, USA). The mRNA gene expression levels were calculated using the comparative (2^-ΔCT^) method where RQ (copies/2 µl of cDNA) = 2 ^^-(Cttreated cells – Ctuntreated cells)^.

**Table 1:** Primers sequences of the studied genes with their respective product sizes and accession numbers.

Gene	Primer sequences	Product size	Accession numbers
β-actin	F: 5′-TGGCACCACACCTTCTACAATGAGC-3′	437	NM_001101.5
	R: 5′-GCACAGCTTCTCCTTAATGTCACGC-3′		
NOTCH1	F: 5′-CCGCCTTTGTGCTTCTGTT-3′	490	NM_017617.5
	R: 5′-TCCTCCTCTTCCTCGCTGTT-3′		
JAG1	F: 5′-GATCCTGTCCATGCAGAACG-3′	436	NM_000214.3
	R: 5′-GGATCTGATACTCAAAGTGG-3′		
HES1	F: 5′-GACAGCATCTGAGCACAGAAATG -3′	374	NM_005524
	R: 5′-GTCATGGCATTGATCTGGGTCAT -3′		

### Statistical analysis

Statistical analysis was carried out with SPSS 22.0 for windows. For comparison of the quantitative variables, Mann Whitney’s test was used to compare between means of different studied groups. Generally, **(p)** values were considered statistically significant at level ≤ 0.05.

## Results

### Cytotoxicity results

The cytotoxicity percentages (%) of CNC (0.1 and 2.55 µM) and DOXO (0.062 and 0.15 µM) concentrations were evaluated against HCC1806 and MDA-MB231 cells, respectively using MTT assay. These concentrations of CNC and DOXO induced significant changes (*P* < 0.05) in cytotoxicity in both cell lines Fig. 1c.

### Cell morphology

**Figure 2:**
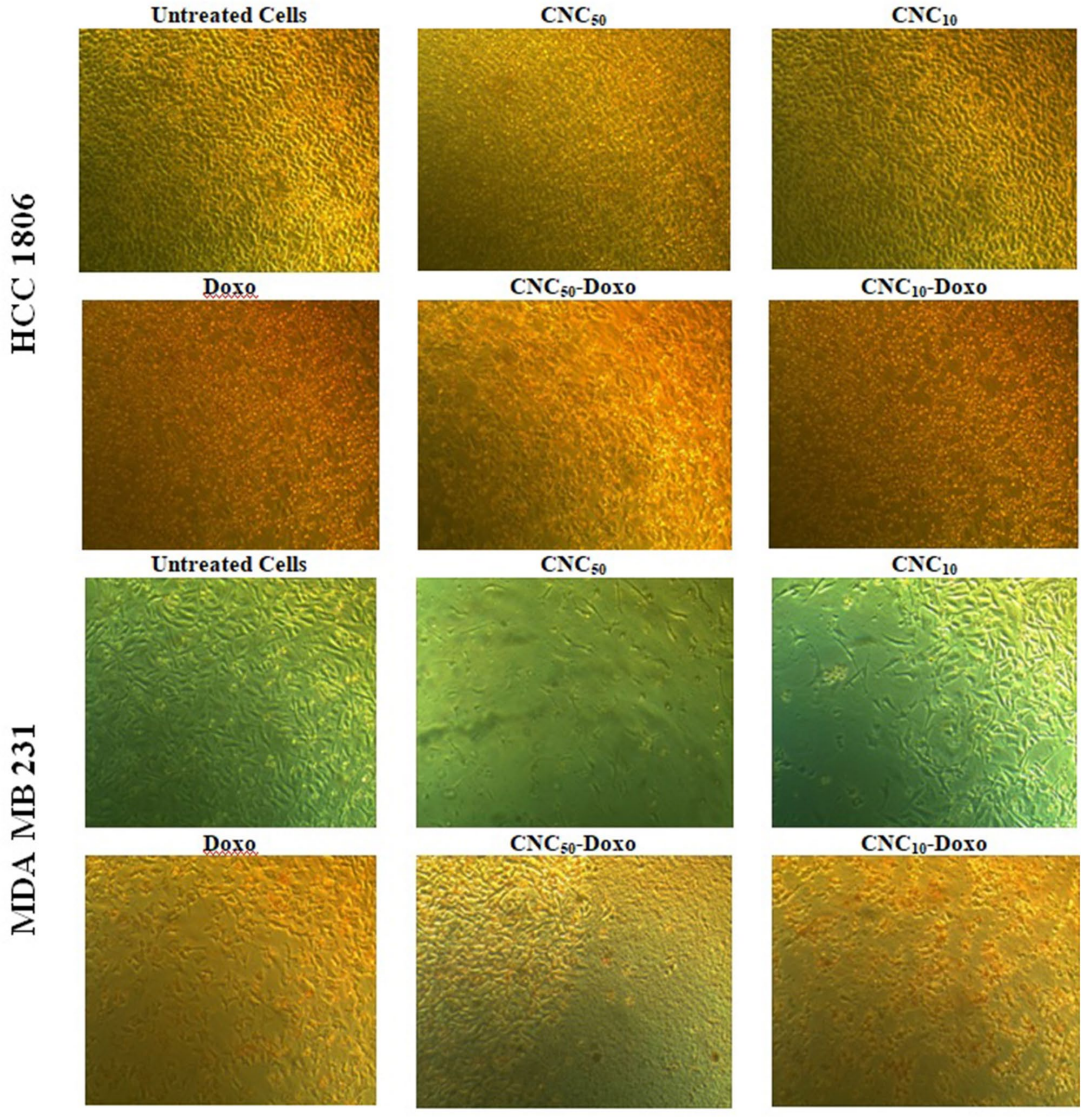
Morphologically alterations of HCC1806 and MDA-MB-231 cells after CNC/Doxo treatment for 24 h. CNC_50_; treated cells with IC_50_ of CNC, CNC_10_; treated cells with 10% IC_50_ of CNC, Doxo; treated cells with IC_50_ of Doxorubicin, CNC_50_-Doxo; treated cells with IC_50_ of CNC and Doxo, CNC_10_-Doxo; treated cells with 10% IC_50_ of CNC and IC_50_ of Doxo. The morphological alterations are relatively more recognizable in MDA-MB-231 cells than in HCC1806 cells.

Visualization of morphological alterations by inverted microscope showed the shrinking and detachment of most of cells after treatment with CNC especially at higher dose. The morphological alterations are relatively more recognizable in MDA-MB-231 cells than in HCC1806 cells Fig. 2.

### Monitoring of autophagy using electron microscopy (TEM)

**Figure 3:**
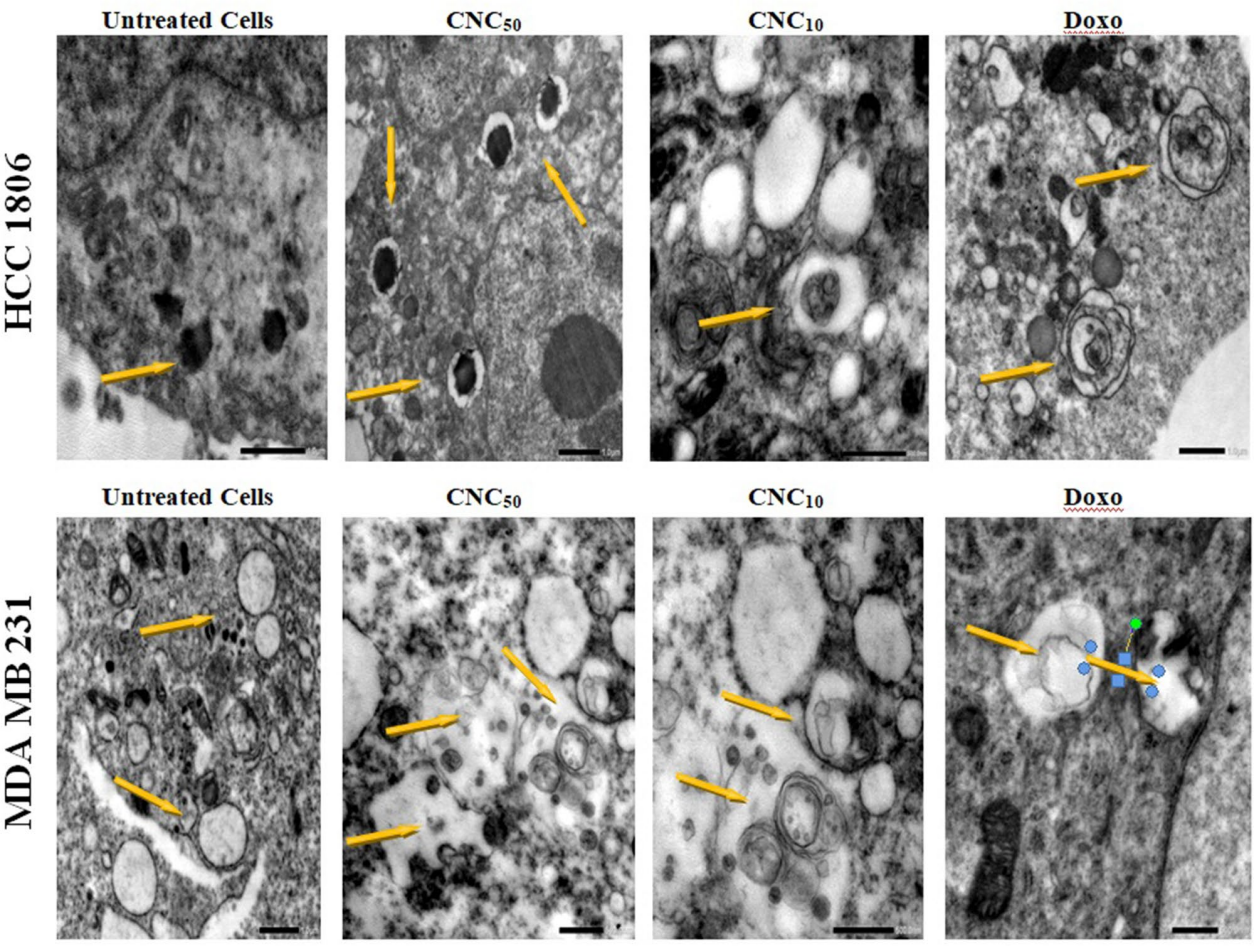
Transmission electron microscope images of HCC1806 and MDA-MB-231 cells, showed the presence of autophagosomes induced by CNC and Doxo treatment for 24 h. CNC50; treated cells with IC_50_ of CNC, CNC10; treated cells with 10% IC_50_ of CNC, Doxo; treated cells with IC_50_ of Doxorubicin, CNC50-Doxo; treated cells with IC_50_ of CNC and Doxo, CNC_10_-Doxo; treated cells with 10% IC_50_ of CNC and IC50 of Doxo. Evidently, treatment of MDA-MB-231 cells with CNC showed a recognizable increase in number of autophagosomes in a dose dependent manner.

TEM images of HCC1806 and MDA-MB-231 cells showed the autophagosomes after treatment with CNC_50_, CNC_10_ and Doxo. In HCC1806 cells, the figure demonstrates a slight decrease in autophagosomes after treatment with either CNC_50_ or CNC_10_. Meanwhile, treatment of MDA-MB-231 cells with CNC showed a recognizable in increase in number of autophagosomes in a dose dependent manner Fig. 3.

### LC3-II detection using flow cytometry

#### Effect of treatment of CNC/Doxo on the number of LC3 positive cells in TNBC cell lines

**Figure 4:**
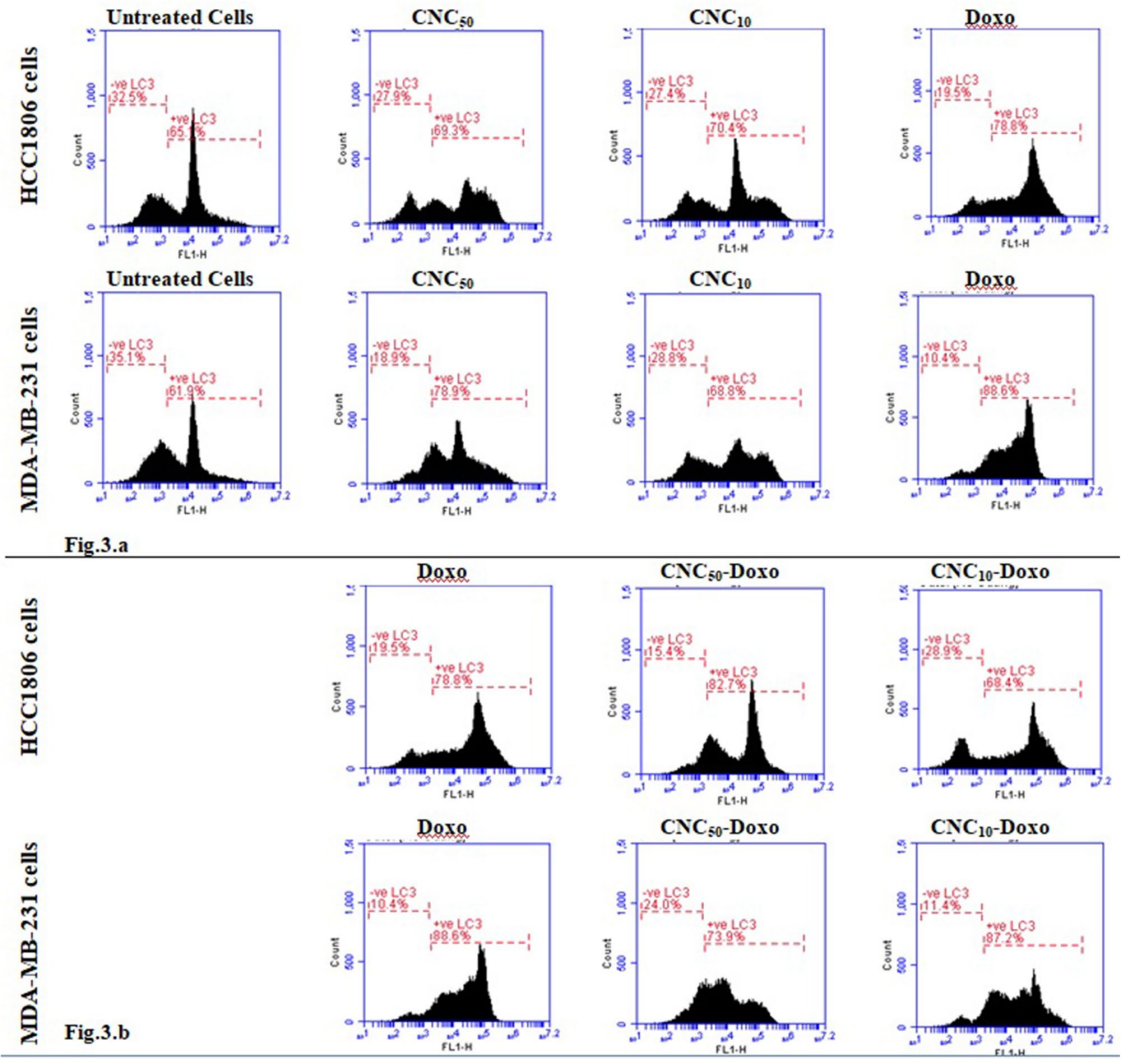
Percent of LC3-II positive cells in HCC1806 andMDA-MB-231 cells after 24 h of treatment; **(a)** under the influence of treatment with CNC or DOXO, **(b)** under the influence of treatment with co-treatment of CNC-DOXO.

**Figure 5:**
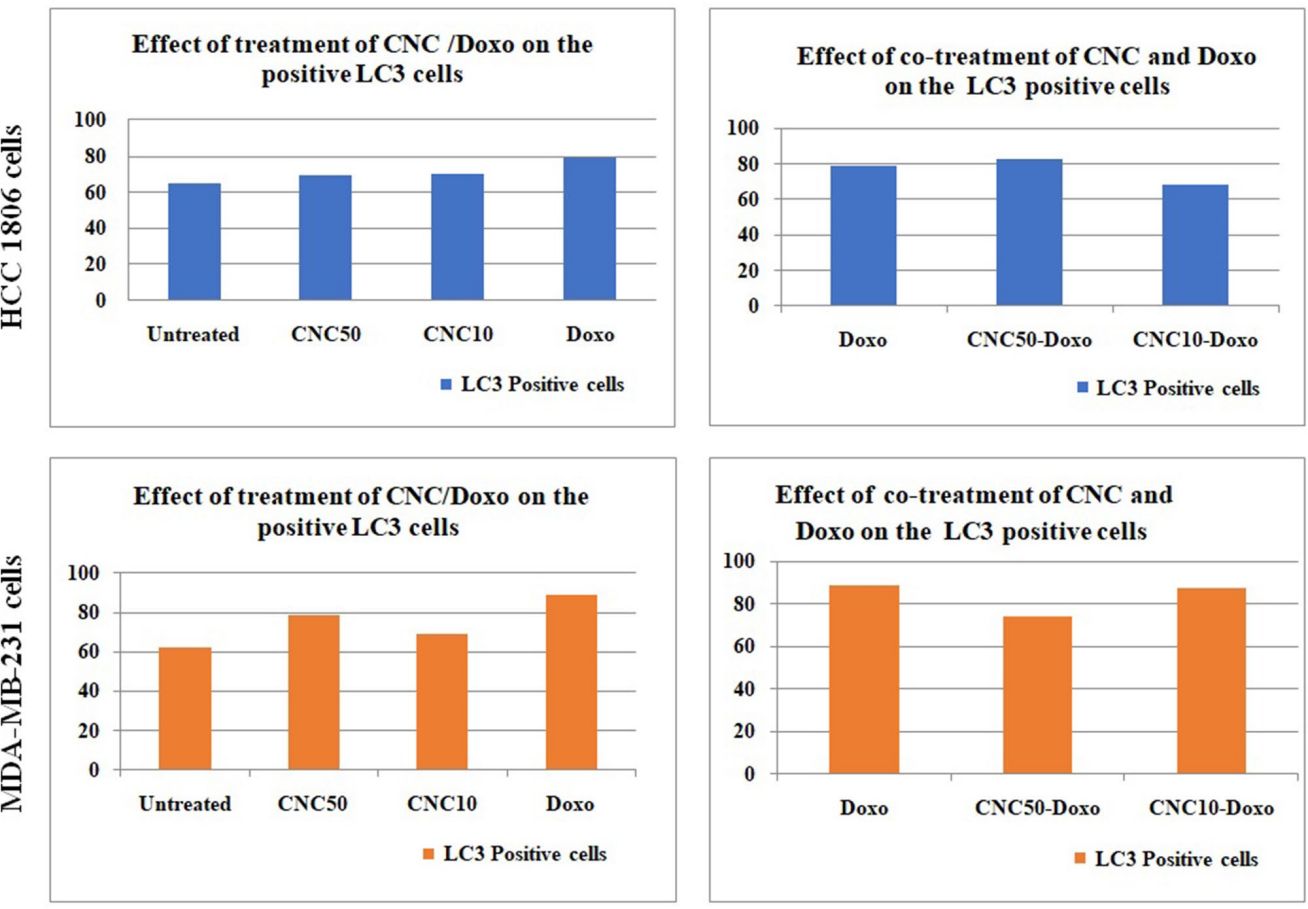
Bar charts showing LC3-II positive cells analysis in HCC1806 and MDA-MB-231 cells under the influence of CNC and Doxo treatments.

Treatment of HCC1806 cells with either CNC_50_ or CNC_10_ slightly increased the number of LC3-II positive cells. Also, treatment with Doxo resulted in an increase in the number of LC3-II positive cells. In MDA-MB-231 cells, treatment with CNC; CNC_50_ or CNC_10_ increased the number of LC3-II positive cells, however, the increase in LC3-II positive cells was more abundant with CNC_50_ than with CNC_10_. These observations are in consistence with the results obtained by TEM. Meanwhile, treatment with Doxo led to greater increase in the number of LC3-II positive cells Figs. 4 and 5

#### Effect of CNC and Doxo co-treatment

Compared to Doxo-treated cells of HCC1806, treating cells with a combination of CNC_50_-Doxo gave rise to minute elevation of the LC3-II positive cells whereas; the combination of CNC_10_-Doxo decreased the number of LC3-II positive cells. With regard to MDA-MB-231 cells, reduced number of LC3-II positive cells was observed upon treatment with a combination of CNC_50_-Doxo, but no change was noticed with CNC_10_-Doxo treatment Figs. 4b and 5

### RT-PCR analysis

#### Effect of CNC/Doxo treatment

**Figure 6:**
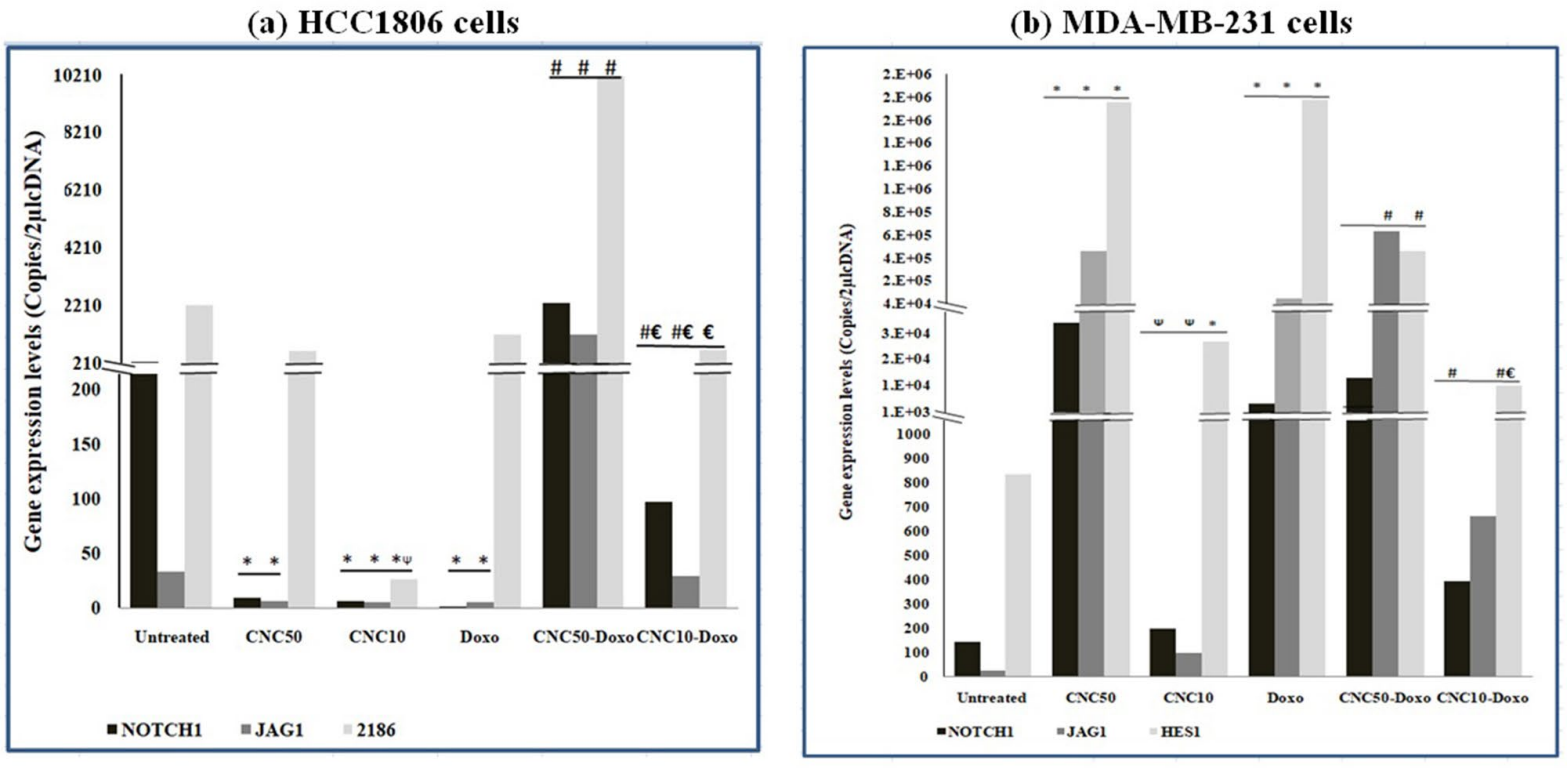
Bar charts showed the statistically analysis (mean ± S.E) of gene expression levels of NOTCH1, JAG1 and HES1 in HCC1806 and MDA-MB231 cells after 24 h of treatments; CNC and DOXO. ^(^*^)^, statistically significant when compared to untreated cells; ^(**Ѱ)**^, statistically significant when compared to CNC_50_ treated cells; ^(**#)**^, statistically significant when compared to Doxo-treated cells; ^(**€)**^, statistically significant when compared to CNC_50_-Doxo-treated cells. *(p)*Values were considered significant at level ≤ 0.05.

Treatment of HCC1806 cells with either CNC_50_ or CNC_10_ resulted in a down regulation in the expressions of NOTCH1, JAG1 and HES1. Sharp and significant down-regulation in the expressions of NOTCH1 and JAG1 were observed in HCC1806 cells when treated with CNC_50_ but was significant for NOTCH1, JAG1 and HES1 upon treatment with CNC_10_ (*p* ≤ 0.05). Also, the expression of HES1 was significantly down regulated by the effect of CNC_10_ when compared to that of CNC_50_ (*p* ≤ 0.05). In contrast to HCC1806 cell line, treatment of MDA-MB-231 cells with CNC_50_ or CNC_10_ resulted in up regulation in the expressions of NOTCH1, JAG1 and HES1. Significant up regulation was observed with the expressions of JAG1 and HES1 *(p* ≤ 0.05). Also, the expression of JAG1 was significantly up regulated by the effect of CNC_50_ when compared to that of CNC_10_ (*p* ≤ 0.05). However, Treatment of both cell lines; HCC1806 and MDA-MB-231, with Doxo induced the same effects as CNC in the corresponding cell line, where in HCC1806 cells, Doxo caused a significant down regulation in the expressions of NOTCH1 and JAG1 (*p* ≤ 0.05). Meanwhile, in MDA-MB-231 cells, it led to a significant up regulation in the expressions of NOTCH1, JAG1 and HES1 (*p* ≤ 0.05) Fig. 6.

#### Effect of CNC and Doxo co-treatment

Compared to Doxo-treated cells, treatment of HCC1806 cells with a combination of CNC_50_-Doxo or CNC_10_-Doxo resulted in a significant up regulation in the expressions of NOTCH1 and JAG1 (*p* ≤ 0.05). Also, a significant up regulation in the expression of HES1 was observed upon treatment with CNC_50_-Doxo (*p* ≤ 0.05). However, down regulation in the expression of HES1 with CNC_10_-Doxo (*p* ≤ 0.05). On the other hand, treating MDA-MB-231 cells with CNC_50_-Doxo caused a significant up regulation in the expression of JAG1 with significant down regulation of HES1 (*p* ≤ 0.05), while CNC_10_-Doxo led to a significant down regulation in the expressions of NOTCH1 and HES1 (*p* ≤ 0.05) Fig. 6.

## Discussion

Two different subtypes of TNBC were utilized in the present study; the acantholytic squamous basal-like (BL) HCC1806 cell line^17^, and the mesenchymal stem-like (MSL) MDA-MB-231 cell line^14^. In consistence with previous report^11^, the IC_50_ values for CNC showed that HCC1806 cells were more sensitive. Additionally, images of the electron micrograph showed that autophagosomes in MDA-MB-231 cells were more obvious than in HCC1806 cells. Furthermore, flow cytometry analysis results showed that the basal level of autophagy in the two cell lines was different. Where, positive cells for LC3-II were 53% and 81.9% in HCC1806 and MDA-MB-231 cells, respectively. All of these observations may contribute to the differences in the malignant behavior and chemosensitivity in both cell lines. Volk-Draper, et al., demonstrated the dramatic differences in sensitivity to chemotherapeutic drugs where HCC1806 cells were more sensitive^14^, while MDA-MB-231 cells are highly resistant to multiple cytotoxic agents and possess stem cell phenotypes^18^. Thus, expectedly the two cell lines will respond differently to the applied treatment.

On the other side, a significant down-regulation in the expressions of NOTCH1 and JAG1 were observed in HCC1806 cells when treated with CNC_50_ whereas, upon treatment with CNC_10_, a significant down-regulation in the expressions of NOTCH1, JAG1 and HES1 were observed. This was associated with increase in LC3-II positive cells denoting elevation in autophagy. These observations are in consistence with the previous reports which claimed that; inhibition of NOTCH1 expression in different types of cancers resulted in induction of autophagy^19,20^ and during stem cell differentiation^21^. The ability of CNC to produce reactive oxygen species (ROS) in concentration dependent manner has been reported^22^. The induced ROS may thus, be involved in the regulation of Notch1 signaling pathway^23^ and promote autophagy^24^. Thus, it could be suggested that the final fate of cells will mainly be CNC concentration dependent. As previously suggested^11^, CNC can be used as an anticancer agent in HCC1806 cells targeting Notch1 signaling pathway but its concentration should be considered. The previous suggestion may be more supported by the observation that CNC almost induced similar effects as that observed upon treating HCC1806 cells with Doxo especially with CNC_10_. However, treatment with Doxo led to down regulation of Notch1 signaling genes; this down regulation was significant with NOTCH1 and JAG1 genes. Regarding its effect on autophagy process, Doxo resulted in an increase in LC3-II positive cells to 78.8% and presence of autophagosomes. These observations are in accordance with previous studies suggested the pro-autophagic effect of Doxo through induction of cytoprotective autophagic flux which diminishes the cytotoxic effects of Doxo^25,26^.

Treating MDA-MB-231cells with CNC_50_ resulted in a significant up regulation in the expression of the Notch1 signaling genes associated with an increase in the number of LC3-II positive cells and the presence of autophagosomes. These are in a line with previous studies showed that Notch hyper-activity favors cell survival and inhibition of apoptosis and may be essential for cell division^25,26^. Meanwhile and with regard to CNC_10_ treatment, it resulted in up regulation of Notch1 signaling genes with pro-autophagic activity. Meanwhile, Tamagnone,*etal*.,2018, have reported that NOTCH1 gene can also act as a tumor suppressor^27^. However, several reports have pointed out to the possibility of Notch1 signaling may have a potent tumor suppressor role in both hematological malignancies and solid tumors and its activation may inhibit cellular growth^28–30^. Generally, Notch1 activation is known to promote cancer development, where as it can also play a tumor suppressor role. Biologically, this is not surprising, in view of the opposite role that Notch1 signaling can exert either a pro-or anti-apoptotic function through multiple mechanisms that are highly cell-and context pendent^23^. As explained before, CNC-anticancer activity might be attributed to ROS production^22^ which has been involved in the regulation of Notch1 signaling pathwa^23^, induction of autophagy and apoptosis through ROS/JNK signaling pathways^24,31,32^ These observations are may provide more evidence for CNC as a promising chemotherapeutic agent in treating MSL subtype of TNBC. As noticed in HCC1806 cells, treating MDA-MB-231cells with Doxo has induced almost similar effects as that produced after treatment with CNC_10_; revealing the pro-autophagic activities of Doxo. Moreover, Doxo led to a significant increase in the expression of Notch1 signaling genes, and according to previous studies, Doxo can strongly increase the expression of Notch1 pathway components in cancer cells^25^.

It should be recognized that Doxo, as an anthracycline, has a high efficacy in treating TNBC but it can result in poor outcomes due to chemoresistance induction^33^. Therefore, complication of Doxo with copper has been reported to be a potential anti-breast cancer agent, having a higher efficacy than the parent drug and could potentially induce apoptosis through increased ROS production^14^. Presently, co-treatment of Doxo and CNC was used to appraise the possibility of sensitizing TNBC to treatment of Doxo. CNC_50_–Doxo resulted in significant up regulation of NOTCH1 signaling genes in HCC1806 cells. Also, it displayed a minor elevation in autophagy. Additionally, CNC_10_–Doxo also gave the same effect up regulating of NOTCH1 and JAG1 and down regulation in HES1 expression with a reduction in autophagy. This difference in the response of autophagy after both treatments may be attributed to the positive correlation found between HES1 expression and autophagy in HCC1806 cells. Accordingly, combined treatment of CNC and Doxo may sensitize the cells to Doxo leading to obvious enhanced antitumor effect that appeared in inhibition of cell viability. This is in consistence with a previous report stated that Notch1 inhibitors; such as CNC in the present case, may potentiate the effect of DNA damaging agents like Doxo in a variety of breast cancer cell lines^34^. With regard to MDA-MB-231 cells, treatment with CNC_50_–Doxo up regulated the expressions of NOTCH1 and JAG1 as well as, down regulated HES1 expression, however, CNC_10_–Doxo caused down regulation of NOTCH1 genes. Besides, CNC_50_-Doxo reduced the number of LC3-II positive cells but, almost no change with CNC_10_-Doxo. Thus, co-treatment of CNC and Doxo may have a substantial improvement in clinical outcome exhibiting an effective strategy for TNBC treatment, as well as, modulation of Notch1 pathway could give enhanced effect and, therefore, might reduce Doxo-induced side effects and resistance in breast cancer treatment^35^.

In conclusion, considering the observed effects induced by Doxo with that produced by CNC may favor the suggestion that CNC may be a promising anti-cancer therapeutic agent for both types of TNBC; BL and MSL subtypes. However, CNC concentration should be taken into consideration. Also, co-treatment of CNC with Doxo can be valid as a potential strategy in treating both TNBC subtypes. Briefly, the produced cytotoxic effects could be imputed to the formation of copper-Doxo complex and its production of ROS. In addition, CNC may facilitate the binding of Doxo to DNA and exert therapeutic effects^36^ besides, the regulation of Notch1signaling pathway by ROS production^23^. All together may explain, in part, the enhanced cytotoxic effects of the co-treatment of CNC and Doxo.

## Data Availability

Data are contained within the article.
