# Author Correction: Influence of copper(I) nicotinate complex on the Notch1 signaling pathway in triple negative breast cancer cell lines

**DOI:** 10.1038/s41598-024-56032-2

**Published:** 2024-03-05

**Authors:** Mohamed A. Abdel‑Mohsen, Asmaa M. Badawy, Morsy A. Abu‑Youssef, Mona A. Yehia, Lobna D. Abou Shamaa, Shymaa Abdullah Mohamed

**Affiliations:** 1 Applied Medical Chemistry Department, Medical Research Institute, Alexandria University, Alexandria, Egypt; 2 Chemistry Department, Faculty of Science, Alexandria University, Alexandria, Egypt; 3 Histochemistry and Cell Biology Department, Medical Research Institute, Alexandria University, Alexandria, Egypt; 4 Immunology and Allergy Department, Medical Research Institute, Alexandria University, Alexandria, Egypt; 5 Molecular Biology Unit, Medical Technology Center and Applied Medical Chemistry Department, Medical Research Institute, Alexandria University, Alexandria, Egypt

Correction to: *Scientific reports* 10.1038/s41598-024-52952-1, published online 30 January 2024

The original version of this Article contained an error in the name of author Shymaa Abdullah Mohamed, which was incorrectly given as Shymaa A. Abdullah.

As a result, the Author contribution section now reads:

“Conceptualization, M.A.A., A.M.B., M.A.A., M.A.Y., L.A.S, and S.A.M.; formal analysis, M.A.A., A.M.B. and S.A.M.; investigation, M.A.A., A.M.B. and S.A.M.; project administration, M.A.A., A.M.B. and S.A.M.; software, M.A.A., A.M.B. and S.A.M.; validation, M.A.A., A.M.B. and S.A.M.; visualization, M.A.A.; writing original draft, A.M.B.; writing review and editing, M.A.A., A.M.B. and S.A.M.; All authors have read and agreed to the published version of the manuscript.”

The original Article has been corrected.

